# A Model for Statewide Educational Outreach to Undergraduates: Implications From a Neuropsychology Outreach Program

**DOI:** 10.59390/001c.146553

**Published:** 2025-12-31

**Authors:** Lynn Schaefer, Hilary Bertisch

**Affiliations:** 1 Department of Psychiatry & Behavioral Sciences, Nassau University Medical Center; 2 Northwell Health

**Keywords:** Outreach, statewide, underrepresented areas, neuropsychology, undergraduate

## Abstract

Clinical neuropsychology is a specialty of psychology that is closely related to neuroscience in that it assesses cognitive and functional brain-behavior relationships across a range of clinical diagnoses. As with many other related disciplines including neuroscience, educational outreach to students about clinical neuropsychology to increase vocational interest is essential to the field. This manuscript examines the feasibility of implementing one statewide educational outreach program for undergraduate students to learn about the specialty of clinical neuropsychology as a possible career path. The current program was developed by the authors of this manuscript, both of whom are clinical neuropsychologists and co-lead a committee for student outreach within a state organization which represents such professionals. As students in metropolitan areas are more likely to have exposure to neuropsychology, the emphasis of our program has been on outreach to university psychology and neuroscience departments, especially in more rural and geographically underrepresented areas of our state. The process of this outreach, the content of the presentations conducted through this program, and descriptive feedback from participants based on qualitative survey data (2023-2025) is presented. Based on the response and successful engagement rate from the colleges and universities that we have outreached, we have determined that our model offers a feasible means to conduct education about possible careers in neuropsychology to undergraduate students statewide. Practical recommendations for neuroscientists, psychologists, and other such professionals interested in initiating and implementing their own outreach programs within their specialties are also provided.

Outreach is an initiative whereby a topic is introduced through brief interaction; audiences are then gained for future education sessions (Thompson et al., 2021). It is not necessarily a long-term commitment; the intention is to spark interest and invite the audience to learn more. Outreach serves as an adjunct to education; it extends the learning environment beyond the classroom or laboratory. Educational outreach to grade school and high school students for the purposes of career exploration has been shown to influence vocational interest (Beale & Williams, 2000; Catrell & Ewing-Taylor, 2009). Specifically, outreach, which is outside the curriculum and based on real-life contexts, has been shown to fulfill basic needs in students including supporting autonomy, feelings of competence, and relatedness (Vennix et al., 2018), such that students had a more positive attitude and motivation towards the subject matter (i.e., Science, Technology, Engineering, Mathematics, “STEM” careers).

Clinical neuropsychology is a specialty of clinical psychology that overlaps with neuroscience in its focus on brain-behavior relationships. Within the field of neuroscience, there have been outreach programs described in the literature that focus on undergraduate universities and K-12 students, but most outreach has used in-person programming in the classroom or during field-trips (Columbia University Neuroscience Outreach, 2025; Gall et al., 2020; Ghani et al., 2024; Neuroscience Institute Public Outreach & Science Education, 2025; Neves et al., 2024; Stevens, 2011; Toledo et al., 2020). Other outreach models have incorporated undergraduate students in neuroscience (Ramadan & Ricoy, 2023; Vollbrecht et al., 2019) or neuroscience and neuropsychology trainees (Rouzer et al., 2023) as trainers to practice communication of scientific ideas, but in this role, they are providing information rather than receiving it. In addition, many major university websites list opportunities for students from kindergarten through post-baccalaureate to participate in live summer programs for neuroscience and other STEM-related careers (MIT Brain & Cognitive Science Outreach, MIT, 2025; Yale Interdepartmental Neuroscience Program, 2025). Since the COVID-19 pandemic, there have also been initiatives to incorporate virtual platforms into educational outreach for neuroscience, particularly geared towards individual schools in remote locations (Oppenheimer et al., 2022; Ramadan & Ricoy, 2023). To the best of our knowledge, however, there have been no known efforts to engage students in educational outreach across broad geographic regions through virtual programming.

Clinical neuropsychologists use their expertise in the assessment and treatment of cognitive disorders in patients across the lifespan with conditions that affect the brain (Schaefer & Bertisch, 2023). Work settings may be in academic medical centers, hospitals, private practice, laboratories and/or universities. Similar to the field of neuroscience, many neuropsychologists also conduct and/or collaborate on a range of research initiatives focusing on brain-behavior relationships. In the U.S., a career in neuropsychology requires a doctoral degree in clinical psychology, including a one-year internship and research dissertation, followed by a two-year fellowship or residency. Following the post-doctoral fellowship, the neuropsychologist can qualify for state licensure and may pursue board certification (Hannay et al., 1998). According to survey responses (ours and those of Schmitt et al., 2024), students are unfamiliar with the specialty of neuropsychology or, if they are, appear to lack information as to the training and the kinds of settings in which neuropsychologists work.

The importance of outreach, advocacy, and public awareness of neuropsychology has been confirmed (Attix & Potter, 2010; Kaseda et al., 2025; Rouzer et al., 2023), although despite its value, outreach is relatively scarce due barriers such as lack of time and funding (Kaseda et al., 2025; Rouzer et al., 2023; Woitowich et al., 2022). Traditionally, neuropsychology outreach has involved public outreach to groups about specific diseases and conditions as well as promoting brain health and cognitive wellness. Neuropsychologists also educate other professionals and potential referral sources about their services and their added value. There is a dearth of information regarding outreach specifically to undergraduate students about the specialty of neuropsychology, There has been some work, however, specifically with historically underrepresented students (Rivera Mindt et al., 2010; Romero et al., 2009). For example, there are organizations, such as New 2 Neuropsychology (N2N 2024; Schmitt et al., 2024), that seek to facilitate greater equity and inclusion in neuropsychology through outreach to historically underrepresented minoritized students. Schmitt et al (2024), through N2N, recently published results of their survey assessing student’s ratings of their knowledge about the field of neuropsychology, how they learned about the field, and perceived barriers to becoming a neuropsychologist. To our knowledge, however, little has been published regarding outreach on a statewide level, including to more geographically rural populations with less access and therefore minimal exposure to the specialty of neuropsychology.

## Under-representation of More Rural Areas within Neuropsychology

It is well-recognized that there is an unequal distribution of medical, mental health, and academic resources in more rural areas as compared to urban areas, which limits access to psychological services in general and to neuropsychological services in particular (Allott & Lloyd, 2009; Huff, 2023; Van Den Broek et al., 2022). This not only impacts patient care in these regions, but also significantly limits access to neuropsychology instructors and mentors for students and limits student opportunities to learn about the unique aspects of the practice of neuropsychology in these regions. Students need to learn about navigating ethics, confidentiality, the limits of competence, and clinical aspects of these populations including higher substance abuse and suicide rates in some rural areas as well as potential social and cultural differences (Huff, 2023). In a 2020 national survey of 1677 doctoral-level neuropsychologists, 81% endorsed practicing in an urban environment, 7% in a rural environment, and 12% in both urban and rural environments (Sweet et al., 2021). Although only about 4% of respondents to this survey indicated that their primary affiliation was a college/university setting, the sample endorsed five hours (SD = 6) of time spent in teaching/training activities per week. Collectively, these data suggest that student exposure to the specialty of neuropsychology may be insufficient to make informed career decisions, and significantly more so in rural or geographically underrepresented areas. Although “rural” has many definitions, we use the definition of rural areas in New York as towns and cities with a population less than 25,000 (Borges et al., 2023).

Since 2020, the COVID-19 pandemic has increased access to healthcare and academic resources to these rural areas through use of virtual platforms (Tsiakiri et al., 2022; Van Den Broek et al., 2022). Students in rural areas now have much greater access to neuropsychology and related seminars and to academic mentorship regardless of the geographic region where the neuropsychologist is located. Now that it is more feasible to provide these educational resources to all students virtually, it is critical for neuropsychologists to do so, both for the benefit of the students and for the long-term growth of our profession. These programs are essential to create a talent pipeline and meet the demands of the profession.

## A Model for Neuropsychology Outreach to Undergraduate Students

The need for outreach about neuropsychology to undergraduate students who may be interested is essential to both the students and to the profession. Although there are likely many methods to conduct such outreach, our initiative was developed through a statewide organization that represents neuropsychologists. In particular, the authors of this manuscript serve as co-leaders of a committee which was developed to educate students at all levels of education about the specialty of neuropsychology. Our purpose was to determine whether it was feasible to create an educational outreach program comprised of a network of colleges and universities throughout the state who refer undergraduates to panel presentations held about careers in clinical neuropsychology.

The New York State Association of Neuropsychology (NYSAN) was established in 2006 as a not-for-profit professional, type 501(C) 6 corporation with the broad intent of acting as a trade organization for neuropsychology in New York. Of most relevance are NYSAN’s objectives of 1) increasing awareness and outreach, 2) education and training, and 3) student support (New York State Association of Neuropsychology, 2024).

As indicated on the website, the mission of the Scholastic Committee of the New York State Association of Neuropsychology “is to educate students of all ages about the field of neuropsychology” (New York State Association of Neuropsychology, 2024). The Scholastic Committee was formed by the authors of this manuscript in 2018 and under this umbrella we began to host educational events for a range of students, with an emphasis on college undergraduates in the contemplative stage of choosing neuropsychology as a career. These events were initially held only in-person in the New York City area, however following the dramatic rise in use of virtual platforms following the COVID-19 pandemic, we began to form ongoing relationships with universities and host these events virtually for undergraduates statewide. Although the New York State Association of Neuropsychology Scholastic Committee provides the ideal support and infrastructure in which to conduct student outreach, our efforts may be replicated through other organized strategies as well.

## MATERIALS AND METHODS

### Identification of Schools

Through simple internet searches we identified every four-year undergraduate college and university in New York State. We then refined the list to include only colleges and universities with an undergraduate psychology and/or neuroscience program, for a total of 126 schools. In some of these schools, the program was called “Psychology, Neuroscience, and Behavior,” “Psychology and Brain Sciences,” “Cognitive and Brain Science,” “Integrative Neuroscience,” “Psychology and Neuroscience,” or was a psychology program with a neuroscience minor. The list was then distributed to the New York State Association of Neuropsychology via our listserv to inform membership about our initiative and with a request to identify any potential contacts they were already familiar with within the departments at each college and university. This request was complemented by further internet searches by the authors to identify additional contacts, preferably individuals in leadership roles, for any departments of which New York State Association of Neuropsychology membership was not directly familiar.

Starting in October 2021, we began to send introductory emails to an initial group of the psychology department contacts, with an emphasis on the schools in more rural areas of the state. The emails provided a brief description of the New York State Association of Neuropsychology Scholastic Committee and our hope to launch virtual educational events about neuropsychology to undergraduates across the state. The contacts were asked whether they would be interested in participating in this initiative by distributing information about upcoming events to their student bodies. If no response was provided within approximately one week, one follow-up email was sent.

**Figure 1.**
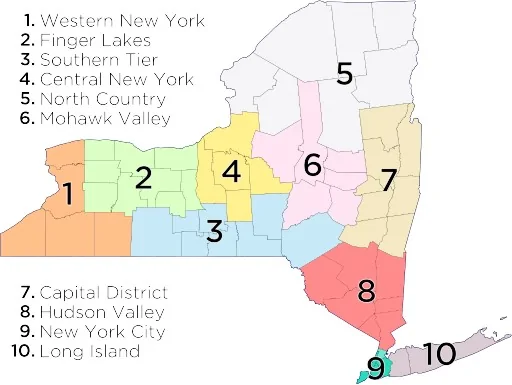
Regions Represented In Our Program. From (https://en.wikipedia.org/wiki/New_York_(state)#/media/File:Map_of_New_York_Economic_Regions.svg)

Approximately 6-8 weeks prior to a planned event, we reached out to new departments on the list to introduce ourselves and allow time for follow-up and connection. The cumulative list of contacts who confirmed interest are sent flyers to distribute before each upcoming educational event. These invitations are generally sent approximately two weeks in advance of the event date. To date, 94 departments across the state have been contacted, twenty-nine have responded with interest, and 20 schools thus far have had students both attend and complete the survey (see below regarding Survey). Figure 1 delineates where in New York State these schools are located, and Table 1 indicates the number of schools in each region that responded with interest. Note that more than half the schools are located “upstate,” which is more rural than “downstate,” indicated by regions 9, 10, and parts of 8. Although some regions contain a city (i.e., New York contains New York City), the catchment area surrounding the city is more rural.

**Table 1.** Number of Interested and Participating Departments by NYS Region

**Region**	**Department(s) Responding with Interest**	
Capital		4
Central		4
Finger Lakes		1
Hudson Valley		3
Long Island		3
Mohawk Valley		1
New York City		6
North Country		2
Southern Tier		2
Western Total		3 29

### Presentations

Our virtual presentations are conducted semi-annually, with dates selected in concordance with the academic calendar, and in one of two formats. One format is a panel discussion, typically conducted in conjunction with our sister organization the New York Neuropsychology Group, whose mission is to “conduct neuropsychological outreach, education, and training among membership and the community” (New York Neuropsychology Group, 2024). These panel discussions are held as two-hour weeknight presentations where, following a review of the slides, neuropsychologists representing various areas of practice describe “a day in the life” of their work. A question-and-answer period is held following these talks. Some links to past outreach events are included at https://www.nyng.org/Outreach.

The second format is a less formal “brown bag lunch,” in which the presenters review an abbreviated set of slides, relate their own experiences in finding careers as neuropsychologists, and then lead an informal discussion and question and answer session. These presentations are held around lunchtime for one hour. This format begins with a variation of a slide presentation outlined in Table 2. These slide presentations were created by the authors, who collectively have several decades of supervisory experience with students at all levels. The authors later integrated this content into their book, “Working with the Brain in Psychology: Considering Careers in Neuropsychology” (Schaefer & Bertisch, 2023).

**Table 2.** Content of Presentations

1. What is neuropsychology? 2. Distinctions and overlaps between psychology, neuroscience, and neuropsychology 3. Patient/client populations that neuropsychologists serve 4. Professional activities where neuropsychologist spend their time (i.e., clinical, research, teaching/supervision, etc.), and what a neuropsychological evaluation entails 5. Related professions (i.e., other mental health providers, neurologists, psychiatrists, Speech and Language Pathologists, neuroscientists, etc.) 6. How to become a neuropsychologist (including training required, first steps and prerequisites) 7. Challenges in the field, including training

### Feedback

To understand the needs of students, the authors offered a feedback survey to participants to learn more about what they already knew and wanted to learn about neuropsychology. Its purpose is to guide content and direction of future educational programs across the state.

Undergraduate students who attended virtual outreach presentations were provided a link or QR code to complete a 30-question survey, which was conducted either in Survey Monkey or Google Forms (see Supplemental Material; demographics of survey respondents in Table 3). The survey was developed by the authors based on questions previously published in psychiatry papers (Goldenberg et al., 2017; Morgan et al., 2024). The survey inquired about prior exposure to neuropsychology, what the student already knows, whether they want to pursue a career in neuropsychology, reasons for wanting to enter the field, possible alternative career paths, and myths/misconceptions about the field. The study was submitted for review to the Institutional Review Board of Nassau University Medical Center and was determined to be exempt (approval #25-294). Of note, this survey is solely qualitative and psychometric validation is outside the scope of this educational initiative, therefore our feedback data are descriptive and are not intended for use in secondary research.

## RESULTS

As of May 2025, we have conducted nine such presentations. Typically, between 10-50 students attend each event. The average is closer to 20 students. To date, 79 students who attended one of four presentations between March 10, 2023 and April 28, 2025 have completed the survey. More than half the schools represented were located in the Capital, Central, North Country, Western, Hudson Valley, Finger Lakes, and Southern Tier regions of New York State. Our sample, fully described in Table 3, ranged in age from 18-37 (mean age = 20.75, median = 20.00) who mostly identified as women (79.7%). Approximately half identified as White (Caucasian; 53.2%), another 13.9% as Hispanic, 12.7% as Black (African American), and 8.9% as Asian. Overall, results indicated that over half (56%) of our participants are upper classmen (i.e., in their junior year or later), and the majority are Psychology majors (60.7%); the second most common major was Neuroscience (27.8%). Approximately half (58.2%) will earn a Bachelor of Arts degree (BA) while 41.8% will earn a Bachelor of Science (BS) degree.

**Table 3.** Demographics of presentation attendee survey respondents (2023–2025)

	**%**
Racial/Ethnic identity	
White	53.2
Hispanic	13.9
Black/African American	12.7
Asian	8.9
Other	11.3
Gender Identity	
Female	79.7
Male	20.3
Transgender	0
Non-binary	0
Prefer Not to Say	0
Grade	
Freshman	22
Sophomore	22
Junior	42
Senior	14
Other	0
Major	
Neuroscience	27.8
Psychology	60.7
Other Science	2.5
Other	9.0
Never Been Exposed to Neuropsychology as a Career	82.3
Intend to Apply to Graduate School for Neuropsychology	39.2
Had a Course in Brain & Behavior	67
Had a Course in Neuropsychology specifically	11.4
School Offered a Major/Concentration in Neuropsychology	50.6
Had Life, Work, or Volunteer Experience with the Brain	50
Interest in Neuropsychology	
Work with and Help People	50.6
Diagnosis and Treatment	22.9
Brain Research	20.2

Interestingly, over a third (39.2%) of participants intended to apply to graduate school for neuropsychology, although 82.3% of students reported never having been exposed to neuropsychology as a career. However, 67% reported having had a course in brain and behavior and 11.4% reported taking a course specifically in neuropsychology. Further, 50.6% reported that their school offered a concentration or major in neuropsychology or similar. Influences outside of school also appear to have played a role in career interest; fully half (50%) of students reported having had life, work, and/or volunteer experience working with the brain. Many respondents were interested in neuropsychology to work with and help people (50.6%) or to be involved in diagnosis or treatment (22.9%), with brain research the third most reported choice (20.2%).

There were many misconceptions, particularly regarding degree required, length of training, and median salary (Table 4). Almost a quarter of students (24%) were unsure of the degree required to become a neuropsychologist, almost half (44.3%) were either unsure of the length of time after earning an undergraduate degree required to become a Board-Certified clinical neuropsychologist or thought it takes four years or less, and 75% did not know the median salary range for an early career Board Certified clinical neuropsychologist working in a hospital setting. Regarding barriers to a career in neuropsychology, although most said they could move for training (65.2% yes; 29.1% “NO”), fully 62% had concerns about student loans. Each of these topic areas is addressed in our presentations.

**Table 4.** Attendee survey respondents’ Misconceptions and Barriers to a Neuropsychology Career (2023–2025)

	**%**
Misconceptions/Misunderstandings	
Unsure of Degree Required	24
Unsure of Length of Time to become Board Certified	44.3
Unsure of median salary range for early career Board-Certified Clinical Neuropsychologist in a hospital setting	75
	
Barriers to a Career in Neuropsychology	
Finances/Student Loans	62
Could you move for Training?	
Yes	65.2
No	29.1
Maybe	5.7

## DISCUSSION

Outreach and education are greatly beneficial to both the student in the contemplative stage of career planning, and ultimately to our profession as neuropsychologists. Any outreach is better than no outreach in this regard, and initiatives can be developed in accordance with the time and resources of the professionals. Using our model, we have successfully engaged with approximately one-third of the colleges and universities with undergraduate psychology and neuroscience departments outreached in our state. This yields a total of 29 schools to date who consistently disseminate information about our bi-annual presentations to their students across every region of New York State, including those areas with an under-representation of neuropsychology and related disciplines. It began with outreach to a small group of contacts from the list we developed, and expanded prior to each new presentation. Thus, the feasibility of our program is evident. With the widespread use of virtual platforms, outreach to colleges and universities even in very rural areas has become much more possible. This has been a boon, particularly as these areas may not have neuropsychologists that could expose the specialty to students, and prevents others from having to travel. Similarly, presentations can range in style from informal “brown bag” lunches to more formal panel presentations. The feedback from our survey results help to tailor our presentations.

### Practical Recommendations for Implementing Outreach Programs

In addition to the existing literature in both neuroscience and psychology outreach, through experience we have learned that outreach initiatives are best if initially communicated among colleagues, especially to limit duplication of efforts and outreach to the same schools or departments about the same topics. For example, depending on the representation of the specialty of psychology within a given state or region, this can be achieved either informally through direct communication, or through more organized larger-scale mechanisms such as a listserv or state psychological association.

Once a target audience is identified, it is recommended that professionals survey perceptions and understanding of their specialty in order to assess gaps in knowledge and misconceptions, adapt outreach accordingly, and continually evaluate the benefits of their outreach efforts. Use of a psychometrically validated tool for this purpose is ideal, but if such a tool does not exist then a qualitative set of focused questions about the area can still provide valuable information.

Although this manuscript describes one model of outreach, other formats are certainly possible. A “train the trainer” model (Hartvigsen et al., 2024) may be used, where a larger group of colleagues may develop and utilize the same format and/or have available the same training materials to implement outreach in more personalized settings with which they may be associated (i.e., local secondary schools in their communities). Within a neuroscience education setting, neuropsychology could be introduced through a guest speaker or through presentation of slides or other shared material, if more formal outreach programs are not available. For example, materials could be downloaded from websites such as the National Academy of Neuropsychology’s Professional Resource page: https://www.nanonline.org/Nanweb24/NAN/Professional_Resources.aspx. On this page, under “Resource Center,” are Educational and Position Papers, including the definition of a neuropsychologist. Short videos are also available for the public on NYSAN’s website (https://www.the-nysan.org/public-videos-on-neuropsychology), and for free with registration at Know Neuropsychology (https://www.knowneuropsych.org). Depending on the preferences of the academic institution, outreach may also be conducted through methods other than planned presentations; examples may include representation at career day events, mentorship programs, or pamphlets with written information made available within high school guidance or undergraduate career counseling departments. Opportunities can be identified through direct outreach to schools, similar to what is described in this manuscript, through alumni, or through online advertisements about relevant academic events.

## Supplementary Material

Supplemental material

## References

[ref-508110] Allott K., Lloyd S. (2009). The provision of neuro-psychological services in rural/regional settings: professional and ethical issues. App Neuropsychol.

[ref-524453] Attix D. K., Potter G. G. (2010). Increasing awareness of clinical neuropsychology in the general public. The Clinical Neuropsychologist.

[ref-508112] Beale A. V., Williams J. C. (2000). The anatomy of an elementary school career day. JCD.

[ref-508113] Borges M., Sipple J. W., Scardamalia B. (2023). State of Rural New York.

[ref-508114] Brain & Cognitive Science Outreach, MIT (2025). https://bcs.mit.edu/community-and-culture-bcs-and-building-46/outreach.

[ref-508115] Catrell P., Ewing-Taylor J. (2009). Exploring STEM career options through collaborative high school seminars. JEE.

[ref-508116] Columbia University Neuroscience Outreach (2025). https://cuno.zuckermaninstitute.columbia.edu/content/programs.

[ref-508118] Gall A. J., Vollbrecht P. J., Tobias T. (2020). Developing outreach events that impact underrepresented students: Are we doing it right?. Eur J Neurosci.

[ref-508119] Ghani N., Baker H., Huntsinger A., Chen T., Familara T.D., Itorralba J.Y., Vanderford F., Zhuang X., Chang C.L., Vo V., Oh E.C. (2024). Science Education for the Youth (SEFTY): A neuroscience outreach program for high school students in southern Nevada during the COVID-19 pandemic. eNeuro.

[ref-508120] Goldenberg M. N., Williams D. K., Spollen J. J. (2017). Stability of and factors related to medical student specialty choice of psychiatry. Am J Psychiatry.

[ref-508121] Hannay H. J., Bieliauskas L., Crosson B. A., Hammeke T. A., Hamsher K. D., Koffler S. (1998). Proceedings of the Houston conference on specialty education and training in clinical neuropsychology. Arch Clin Neuropsychol.

[ref-508122] Hartvigsen S.C., Burnett T., Fox C.M., Matney C.J., Pham D., Smiley C.E., Shah A.P. (2024). Mini-Symposium: Training the trainers of the next generation of neuroscience advocates. J Undergrad Neurosci Educ.

[ref-508123] Huff C. (2023). Preparing for practice in rural communities: Supervisors and trainees can hone specific skills to deliver more ethical and effective practice in rural communities. APA Monitor on Psychology.

[ref-508124] Kaseda E. T., Maietta J. E., Evangelista N. D., Kapoulea E. A., Fernandes M. A., Ellison R. L. (2025). Advocacy and leadership in neuropsychology doctoral education: A developmental integration of perspectives on training. J Clin Exp Neuropsychol.

[ref-508127] Morgan L. J., Finn G. M., Tiffin P. A. (2024). Are efforts to recruit to psychiatry closing the stable door after the horse has bolted? Knowledge and attitudes towards a career in psychiatry amongst secondary (high) school students: a UK-based cross-sectional survey. J Ment Health.

[ref-508128] Neuroscience Institute Public Outreach & Science Education (2025). https://med.nyu.edu/departments-institutes/neuroscience/neuroscience-institute-the-community/public-outreach-science-education.

[ref-508129] Neves B.-H.S.D., Martini V.Á., Fantti M.F., Mello-Carpes P.B. (2024). Long-term impact of neuroscience outreach interventions on elementary students' knowledge. Adv Physiol Educ.

[ref-508130] New 2 Neuropsychology (2024). N2N: New 2 Neuropsychology.

[ref-508131] New York Neuropsychology Group (2024). New York Neuropsychology Group: Specialty Committees.

[ref-524454] New York State Association of Neuropsychology (2024). About NYSAN.

[ref-508134] Oppenheimer K. E., Salig L. K., Thorburn C. A., Exton E. L. (2022). Taking language science to zoom school: Virtual outreach to elementary school students. Lang Linguist Compass.

[ref-508135] Ramadan B., Ricoy U. M. (2023). The NEURON Program: Utilizing low-cost neuroscience for remote education outreach. JUNE.

[ref-508136] Rivera Mindt M., Byrd M., Saez P D., Manly J. (2010). Increasing culturally competent neuropsychological services for ethnic minority populations: A call to action. TCN.

[ref-508137] Romero H. R., Lageman S. K., Kamath V., Irani F., Sim A., Suarez P., Manley J. J., Attix D. K., The Summit participants (2009). Challenges in the neuropsychological assessment of ethnic minorities: Summit proceedings. TCN.

[ref-508138] Rouzer S.K., Kalinowski L.M., Kaseda E.T. (2023). The importance of promoting scientific advocacy & outreach for trainees. Neuropsychopharmacology.

[ref-508139] Schaefer L. A., Bertisch H. C. (2023). Working with the Brain in Psychology: Considering Careers in Neuropsychology.

[ref-508140] Schmitt T. R., Van Patten R., DesRuisseaux L. A., Yurievna Gotra M., Hewitt K. C., Peraza J., Tan A., Votruba K. L., Bellone J. A., Block C., Talbert L. D., Ray C., Kaseda E. T., Owens R., Martinez M. N., Persad C. C., Stringer A. Y. (2024). New2Neuropsychology (N2N): An organization to promote diversity, equity, and inclusion in neuropsychology. TCN.

[ref-508141] Stevens C. (2011). Integrating community outreach into the undergraduate neuroscience classroom. JUNE.

[ref-508142] Sweet J. J., Klipfel K. M., Nelson N. W., Moberg P. J. (2021). Professional practices, beliefs, and incomes of U.S. neuropsychologists: The AACN, NAN, SCN 2020 practice and "salary survey". J Clin Neuropsychol.

[ref-508143] Thompson V. L. S., Ackermann N., Bauer K. L., Bowen D. J., Goodman M. S. (2021). Strategies of community engagement in research: Definitions and classifications. TBM.

[ref-508144] Toledo M. A., Koochak N., Gupta A., López L. N., Nieri T., Currás-Collazo M. C. (2020). Interactive student-centered neuroscience workshops for sixth graders enhance science knowledge and education attitudes. JUNE.

[ref-508145] Tsiakiri A., Koutzmpi V., Megagianni S., Toumaian M., Geronikola N., Despoti A., Kanellopoulou S., Arampatzi X., Margioti E., Davila A., Zoi P., Kalligerou F., Liozidou A., Tsapanou A., Sakka P. (2022). Remote neuropsychological evaluation of older adults. Appl Neuropsychol Adult.

[ref-508146] Van Den Broek S. R., Bagot K. L., Arthurson L., Cadilhac D. A., Stolwyk R. J. (2022). Investigating clinician experiences of teleneuropsychology service implementation within rural inpatient rehabilitation settings: A mixed method approach. Arch Clin Neuropsychol.

[ref-508147] Vennix J., den Brok P., Taconis R. (2018). Do outreach activities in secondary STEM education motivate students and improve their attitudes towards STEM?. Int J Sci Educ.

[ref-508149] Vollbrecht P. J., Frenette R. S., Gall A. J. (2019). An effective model for engaging faculty and undergraduate students in neuroscience outreach with middle schoolers. JUNE.

[ref-508150] Woitowich N. C., Hunt G. C., Muhammad L. N., Garbarino J. (2022). Assessing motivations and barriers to science outreach within academic science research settings: A mixed-methods survey. Front Commun.

[ref-508151] Yale Interdepartmental Neuroscience Program (2025). Outreach.

