# Supplementary material for: A Model for Statewide Educational Outreach to Undergraduates: Implications From a Neuropsychology Outreach Program
Source: J Undergrad Neurosci Educ. 2025 Dec 31;24(1):66–74. doi: 10.59390/001c.146553 (PMC13127486; doi:10.59390/001c.146553)
Supplement: Supplemental material [file junejournal_2025_24_1_146553_308451.pdf]

NYSAN Educational Outreach Undergraduate Survey

Insights into how and when students get introduced to and chose Neuropsychology as a career

Not shared

* Indicates required question

New York State

NYSAN

Association of Neuropsychology

What university or college are you currently attending? *

Your answer

What year are you in? *

- ○ Freshman
- ○ Sophomore
- ○ Junior
- ○ Senior
- ○ Other:

Age *

Your answer

With what gender do you identify? *

- ○ Male
- ○ Female
- ○ Non-binary
- ○ Prefer not to say
- ○ Other:

With what race/ethnicity do you most identify? *

- White
- Black
- Hispanic
- Asian
- Prefer not to answer
- Other:

What is your college major? *

- Psychology
- Neuroscience
- Biology
- Other social science
- Other science
- Other:

Are you planning on applying to graduate school in neuropsychology? *

- Yes
- No
- Unsure

If so, what is your primary interest in going into neuropsychology? *

- ○ Interested in working with/helping patients
- ○ Research
- ○ Diagnosis and treatment
- ○ Teaching
- ○ Other:

Rank how important the following factors are in your choosing a career (1=least; 6=most) *

	1	2	3	4	5	6
Lifestyle and work-life balance	○	○	○	○	○	○
Prestige	○	○	○	○	○	○
Ability in the field	○	○	○	○	○	○
Interest in the field	○	○	○	○	○	○
Financial reward	○	○	○	○	○	○
Want to help people	○	○	○	○	○	○

If you didn't go into neuropsychology, what alternative career(s) have you considered: *

- [ ] Medicine (Psychiatry)
- [ ] Medicine (non-Psychiatry)
- [ ] Other medical, not medicine (PA, nursing)
- [ ] Psychology (not Neuropsychology)
- [ ] Other mental health
- [ ] Veterinary
- [ ] Dentist
- [ ] Pharmacy
- [ ] Biological or other life science
- [ ] Rehab (PT, OT, SLP)
- [ ] Business
- [ ] Law
- [ ] Other:

Have you ever been exposed to neuropsychology as a career? *

- ○ Yes
- ○ No

If so, how

Your answer

If you go into neuropsychology, what do you foresee as your primary work setting? *

- Private practice
- Hospital
- Research
- Teaching
- Forensic
- Don't know
- Other:

If you go into neuropsychology, and see patients, what do you foresee as your primary patient population? *

- Neurological
- Psychiatric
- Rehabilitation
- Forensic
- Don't know

If you go into neuropsychology, and see patients, what do you foresee as your primary patient age? *

- ◯ Adult only
- ◯ Pediatric only
- ◯ Lifespan
- ◯ Don't know

Will you, or have you, earned a BA or BS? *

- ◯ BA
- ◯ BS
- ◯ Other:

Which of the following hobbies have you engaged on campus *

- ☐ Sports
- ☐ Political
- ☐ Newspaper/website/radio
- ☐ Outdoor/nature
- ☐ Religious
- ☐ Music/art/performing arts
- ☐ Other:

Do you have a parent or other family member who is a neuropsychologist? *

- [ ] Yes
- [ ] No

Do you have a parent or other family member who is a psychologist (but not a neuropsychologist)? *

- [ ] Yes
- [ ] No

Do you have a parent or other family member who is in medicine or other healthcare (not mental health)? *

- [ ] Yes
- [ ] No

Do you have a parent or other family member who is in another mental health field (MHC, MSW, MFT, etc)? *

- [ ] Yes
- [ ] No

Do you have any life, work, and/or volunteer experience working with brain conditions? *

- Yes
- No

Have you ever had a course in brain and behavior (i.e., physiological psychology or behavioral neuroscience)? *

- Yes
- No

Have you ever had a course in neuropsychology specifically (not just physiological psychology or behavioral neuroscience)? *

- Yes
- No

Does your university/college offer a concentration or major in neuropsychology (or similar - i.e., brain and behavior, integrative neuroscience, biopsychology, psychobiology)? *

- Yes
- No
- Unsure

If you go into neuropsychology, what do you foresee as your path? *

- ○ Clinical
- ○ Research
- ○ Teaching
- ○ Don't know
- ○ Other:

Do you know the degree needed to become a neuropsychologist? *

- ○ MD
- ○ PhD/PsyD
- ○ MA/MS
- ○ Don't know
- ○ Other:

Do you know how many years after undergrad (on average) to become a board-certified neuropsychologist? *

- ○ Two
- ○ Four
- ○ Five
- ○ Eight
- ○ Twelve
- ○ Don't know

Do you know the median salary, in US dollars, for a board-certified neuropsychologist working in a hospital setting (with less than 10 years of experience)? *

- 50,000-60,000
- 60,000-80,000
- 80,000-100,000
- 100,000-130,000
- 130,000-150,000
- 150,000-180,000
- Over 180,000
- No idea

Would you be able to move (i.e., live in a different state, even temporarily) for training? *

- Yes
- No
- Maybe

Would student loans be a concern for training? *

- Yes
- No

Submit

Clear forr

Never submit passwords through Google Forms.
